# The Effect of an Arthroscopic Orthopaedic Procedure on a Professional Tennis Player’s Career

**DOI:** 10.7759/cureus.5654

**Published:** 2019-09-14

**Authors:** Andrew George, Matthew D Saltzman, Wellington K Hsu

**Affiliations:** 1 Orthopaedic Surgery, Northwestern University Feinberg School of Medicine, Chicago, USA

**Keywords:** tennis, surgery, outcomes

## Abstract

Orthopaedic injuries can significantly impact the careers of professional tennis players. It is currently unknown how professional tennis players fare after arthroscopic surgery. For the purpose of this study, players ranked in the Association of Tennis Professionals (ATP) and Women’s Tennis Association (WTA) who underwent arthroscopic surgery of any joint between 1996 and 2016 were identified through a well-established, previously published protocol of injury reports and public archives. Performance statistics both before and after surgery, time to return (TTR) to play, and career length following surgery were collected for each player. Statistical analysis was performed with significance accepted at a probability value (p) of <0.05. A total of 55 (39 males and 16 females) players met the inclusion criteria (shoulder, n = 15; elbow, n = 15; wrist, n = 13; hip, n = 12). The average age of the players at the time of surgery was 25.8 (±4) years, and the average career length before surgery was 8.4 (±4) years. Tennis players who underwent arthroscopic shoulder surgery experienced a longer TTR to play (279 days, p <0.01), as well as a greater decline in their rankings, both in the first and second years postoperatively (p <0.01 and p = 0.01, respectively), compared to all other surgical cohorts. Players who underwent surgery on the elbow, wrist, and hip had no significant decline in the ranking by the second postoperative year. There were no significant differences between genders. This study represents the largest database of professional tennis players who have undergone arthroscopic surgery and may allow physicians to provide evidence-based recommendations about expectations after surgical treatment.

## Introduction

The physical demands of professional tennis players often lead to a high rate of musculoskeletal injuries [1-3]. Modern racquets have enabled players to generate ball velocities upwards of 140 mph, resulting in a substantial amount of force transmitted through the body at contact with the ball [4, 5]. Furthermore, the force used to generate these ball velocities flows through a kinetic chain: from the lower extremities through the trunk, and ultimately into the shoulder, elbow, and wrist [6]. Structural damage and injury to ligamentous, tendinous, and labral structures can occur as a result of repetitive forces experienced by these joints. The emphasis on ball velocity, along with very demanding professional tour schedules, has been associated with an increase in injuries over the past 20 years, resulting in a higher rate of injury per playing hour in tennis compared to other sports such as rugby and basketball [1-3].

Arthroscopic surgery is often considered for structural injuries when conservative treatment fails. Prior studies have demonstrated favorable outcomes after surgery in recreational tennis players and in other overhead athletes such as baseball pitchers [7-12], but few studies have critically examined outcomes in professional tennis players. Since professional tennis players have substantially greater physical demands than recreational athletes, their return-to-play and career-length outcomes may vary. While small case series have reported limited performance statistics after individual procedures [13, 14], their impact on the professional player’s career in terms of short-, medium-, and long-term consequences is unknown. It is also unclear how outcomes differ depending on which joint is affected. This information may be helpful for players, coaches, and physicians assessing the potential impact of an injury and surgery on a professional tennis player’s career.

In our study, we sought to assess and compare performance-based outcomes following arthroscopic surgery in both male and female professional tennis players by utilizing important measures including world ranking, recovery time, and career length.

## Materials and methods

Using a previously published methodology that involved the review of public archives [15-20], we identified professional singles tennis players ranked in the Association of Tennis Professionals (ATP) and Women’s Tennis Association (WTA) who underwent arthroscopic surgery of any joint between 1996 and 2016. Institutional review board approval was not required for this study as it used public data only. Online databases including NewsBank (NewsBank Inc., Naples, FL), the Infosys ATP Player Database, and the WTA Player Database were explored using search terms such as “ATP injury”, “WTA injury”, “professional tennis injury”, “arthroscopic surgery”, “shoulder surgery”, “elbow surgery”, “wrist surgery”, “hip surgery”, “knee surgery”, and “ankle surgery”. Players were deemed to meet the inclusion criteria if reports of the injury, surgery, and date of surgery were corroborated by at least two independent sources of information, including press releases, newspaper archives, and injury reports. Players were excluded if they had undergone concurrent procedures, had received only non-operative management, or had undergone revision procedures within 3 years of surgery. Those players whose reports contained conflicting information from different sources were also excluded. In addition, players who primarily played in doubles matches were excluded, due to potential variability in doubles partner skills and performance. The shoulder, elbow, wrist, and hip cohorts were included, as their sample sizes met the threshold for statistical analysis using a one-way independent analysis of variance (n = 10, a = 0.05, b = 0.2).

Player variables including age, sex, body mass index (BMI), hand dominance, career length before surgery, date of surgery, date of the first match played after surgery, and career length after surgery were collected for each athlete. To assess performance, we collected year-end singles ranking in the preoperative year, as well as the rankings in the first and second years postoperatively. In addition, we collected the number of aces per service game for male players (not available for female players) using the Infosys ATP Player Database. The preoperative year was defined as the year immediately preceding the injury-shortened year. Postoperative years one and two were defined as the first and second years after the athlete returned post-surgery, respectively The athlete’s preoperative performance was used as a baseline to compare with postoperative performance. We did not include data beyond the postoperative year two due to the potential effects of confounding variables such as unrelated injuries, age, and talent. Finally, time to return (TTR) was defined as the number of days between surgery and the first match played postoperatively.

Statistical analysis was performed using Statistical Analysis System (SAS; version 9.4; SAS Institute, Cary, NC). Cohorts of players undergoing arthroscopic shoulder, elbow, wrist, and hip surgery were compared using analysis of variance (ANOVA). Each athlete served as his/her own control with a two-tailed paired t-test used to evaluate changes in performance after surgery. Statistical analysis was performed with significance accepted at a probability value (p) of <0.05.

## Results

A total of 55 (39 males and 16 females) professional tennis players who underwent arthroscopic surgery between 1996 and 2016 were included (shoulder, n = 15; elbow, n = 15; wrist, n = 13; hip, n = 12) (Table 1). Although reported diagnoses leading to surgical intervention included rotator cuff tear, labral tear of the shoulder, bone spurs of the elbow, ligament damage of the wrist, and femoroacetabular impingement of the hip, this information was not reliably reported for several players with the methodology used.

**Table 1:** Demographics of professional tennis players before surgery. Reported as “mean (standard deviation)”, *p = 0.002.

	Sample size (n)	BMI (kg/m^2^)	Age at injury (y)	Playing experience (y)
Overall	55	22.7 (1.80)	25.8 (4.01)	8.4 (4.01)
Shoulder	15	22.1 (2.17)	26.2 (3.78)	8.9 (3.74)
Elbow	15	23.0 (1.09)	26.0 (4.98)	8.3 (5.01)
Wrist	13	22.3 (1.87)	25.4 (3.34)	8.2 (3.02)
Hip	12	23.7 (1.64)	25.4 (4.05)	8.1 (4.33)
Male	39	23.5 (1.03)*	26.3 (4.16)	8.4 (4.34)
Female	16	20.8 (1.90)	24.5 (3.40)	8.4 (3.21)

All of the shoulder and elbow procedures that met the inclusion criteria were performed on the players’ dominant arms (shoulder: 12 right-handed, 3 left-handed; elbow: all right-handed). Eleven of the 13 wrist surgeries were on the players’ non-dominant wrists (11 right-handed, 2 left-handed). Significantly, all players in the wrist cohort were known to utilize a two-handed backhand. Ten of the 12 hip surgeries were on the right hip, and all players in this cohort were right-handed.

The sole demographic difference was a significantly higher BMI in male players compared to female players (p <0.01) (Table 1). The average age at the time of surgery was 25.8 (±4) years, and the average professional career length before surgery was 8.4 (±4) years (Table 1). There were no significant differences between joint cohorts in player age or career length before surgery (p = 0.93 and p = 0.95, respectively). Furthermore, there were no significant differences between cohorts in year-end singles ranking for the preoperative year (Table 2). Recovery time was significantly longer following shoulder surgery compared to the elbow, wrist, and hip surgery (p <0.01) (Table 3).

**Table 2:** World ranking before surgery. SD: standard deviation.

	Sample size (n)	Avg. ranking	SD
Overall	55	63	76
Shoulder	15	72	71
Elbow	15	44	44
Wrist	13	56	70
Hip	12	83	112
Male	39	70	79
Female	16	48	67

**Table 3:** Time to return to sport following surgery. TTR: time to return, *p <0.01. SD: standard deviation.

	Sample size (n)	Avg. TTR (days)	SD
Overall	55	186	117
Shoulder	15	279*	100
Elbow	15	142	117
Wrist	13	148	78
Hip	12	163	117
Male	39	180	119
Female	16	202	115

**Figure 1:**
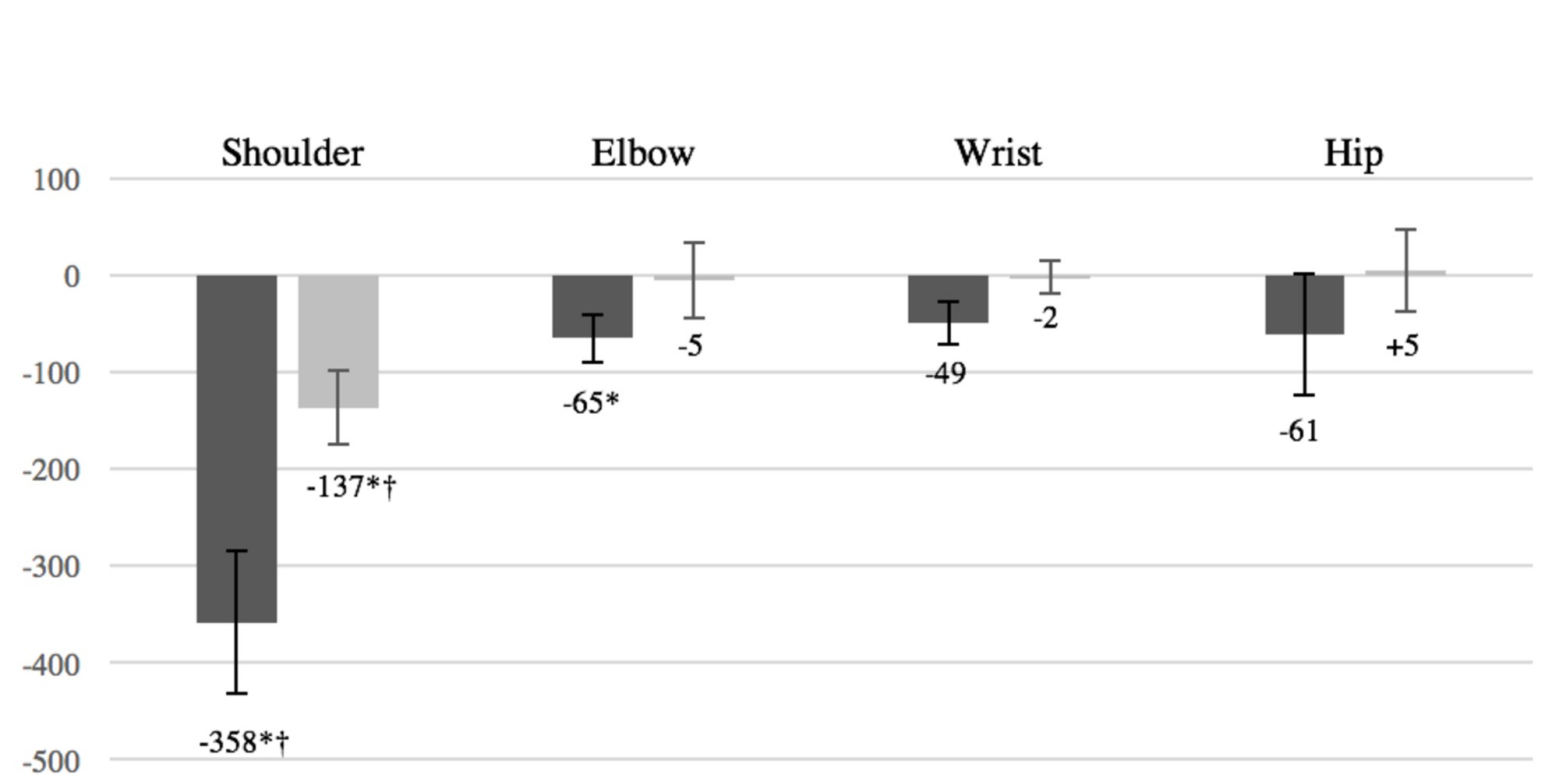
Change in ranking after surgery. Dark gray: change in ranking from preinjury year to return year one. Light gray: change in ranking from preinjury year to return year two.

Athletes who underwent arthroscopic shoulder or elbow surgery had a significant decline in ranking after the first postoperative year of play (p <0.01 and p = 0.03, respectively) (Figure 1). While the elbow cohort returned to baseline ranking in the second postoperative year, players who underwent arthroscopic shoulder surgery continued to have significantly lower rankings compared to the baseline (p <0.01). Furthermore, the shoulder-surgery cohort demonstrated a significantly greater decline in ranking compared to all other cohorts in both the first and second years postoperatively (p <0.01 and p = 0.01, respectively) (Figure 1). From a performance-metric perspective, there was a significantly greater decrease in aces per service game in the shoulder cohort compared to all other cohorts in the first year postoperatively (shoulder: 0.20 aces-per-game decrease; elbow: 0.01 aces-per-game decrease; wrist: 0.08 aces-per-game increase; hip: 0.03 aces-per-game decrease; p = 0.04).

After appropriate recovery time, athletes who underwent arthroscopic wrist or hip surgery demonstrated no significant change in ranking, at either time point. While every player in the shoulder cohort experienced a decline in ranking after the first postoperative year, a few players in each of the other cohorts showed improvement in the ranking (elbow: 4 players; wrist: 2 players; hip: 3 players).

Out of the 55 tennis players who underwent surgery, 30 are currently active in the professional circuit. Considering the data available for those who retired (shoulder, n = 6; elbow, n = 6; wrist, n = 6; hip, n = 7), the average career length after surgery was 5.7 (±2.6) years. And there was no significant variation on this metric depending on the location of surgery either (shoulder: 5.5 years; elbow: 6.2 years; wrist: 7.1 years; hip: 3.8 years; p = 0.14). There were no significant differences in any postoperative measures between genders. 

## Discussion

Tennis players faced with musculoskeletal injuries often undergo arthroscopic surgery with the goal of returning to their baseline level of performance. In comparison to recreational athletes, professional tennis players must return to a significantly more demanding level of play in a safe and timely manner. Since the impact of injury and the recovery time are much more critical in this patient group, athletes and their medical teams must be aware of the impact of surgical treatments on expected postoperative outcomes.

The data in our study suggest that professional tennis players who underwent arthroscopic shoulder surgery experienced the longest TTR and the greatest decline in postoperative performance. This is consistent with the data reported by Young et al., who found that arthroscopic shoulder surgery in female tennis players (n = 8) was associated with a prolonged and incomplete return to play [14]. There are a number of potential explanations for these findings. One explanation is that the shoulder is a critical joint in the production of all tennis strokes, especially the serve [21]. Significantly, all athletes in the shoulder cohort underwent surgery on their dominant arm. Furthermore, prior research in joint biomechanics has demonstrated that dominant-shoulder rotation is the greatest contributor to serve velocity in elite tennis players [22-24]. Thus, arthroscopic shoulder surgery may be associated with a reduction in the serve velocity, a critical component of performance in professional players. This is consistent with our results that male players in the shoulder cohort experienced a significantly greater drop in aces per service game compared to all other cohorts. The decline of ranking in the shoulder cohort may also be related to the longer TTR since time away from the sport could negatively impact performance and ranking.

Conversely, players who underwent elbow, wrist, and hip surgeries experienced significantly better outcomes. Athletes who underwent arthroscopic elbow surgery demonstrated a significant decline in ranking in their first postoperative year. However, while the shoulder cohort continued to have lower rankings after the second year of return, the elbow cohort returned to baseline performance levels after the second year. Of note, the majority of wrist operations were performed on the non-dominant wrist, which is consistent with previous literature reporting a higher prevalence of injuries in the non-dominant wrists of players with two-handed backhands [25]. Similarly, our findings in the hip cohort suggest that the hip on the dominant side is at a greater risk for injury and the need for surgical treatment. This may be due to the role of “back leg drive” in the initiation of trunk rotation for the serve and forehand, which exerts substantial stress on the dominant hip [21, 26].

The average age and career length before surgery were very similar across cohorts (Table 1). This finding may suggest that the pathology leading to arthroscopic treatment was a chronic, wear-and-tear mechanism as opposed to an acute injury leading to a structural lesion. Further studies may lead to training protocols designed to strengthen these joints prior to this time threshold in a professional tennis player’s career, in order to avoid future surgical procedures.

Although this study utilized a previously published protocol, several important limitations remain. Details regarding injury diagnosis, radiographic findings, and exact procedure performed were not available for all players, which limited the conclusions we could make. We attempted to compensate for this limitation by utilizing each player as his/her own control, as well as by comparing cohorts as a whole. However, we were unable to make conclusions regarding the effect of the specific type of arthroscopic surgery. It is possible that the performance after debridement is different than that after repair. In addition, changes in ranking may have been influenced by a variety of other factors, including first-round exits, time off from surgery, and opponent skill level. Finally, it is possible that successful, newsworthy players had a higher chance of meeting the inclusion criteria given our methodology of using public archives. However, there was still a considerable gulf among the ranking of the players whose pre-surgery data we analyzed (Table 2), which indicates that the implications of this study are not limited to top-ranked players only.

## Conclusions

Professional tennis players are predisposed to a variety of musculoskeletal injuries that may require arthroscopic surgery, including those of the shoulder, elbow, wrist, and hip. The results of this study suggest that arthroscopic shoulder surgery is associated with a prolonged TTR to play and a significant decline in ranking after the first and second years of return from surgery. To the best of our knowledge, this study represents the largest database of professional tennis players who have undergone arthroscopic surgery and may allow physicians to provide evidence-based recommendations about expectations after surgical treatment.
